# Primary Screening of the Bioactivity of Brackishwater Cyanobacteria: Toxicity of Crude Extracts to *Artemia salina* Larvae and *Paracentrotus lividus* Embryos

**DOI:** 10.3390/md803471

**Published:** 2010-03-05

**Authors:** Viviana R. Lopes, Nuria Fernández, Rosário F. Martins, Vitor Vasconcelos

**Affiliations:** 1 LEGE/CIIMAR/CIMAR_LA-Laboratory of Ecotoxicology, Genomic and Evolution-Centre of Environmental and Marine Research, University of Porto, Rua dos Bragas 289, 4050-123 Porto, Portugal; 2 Department of Biology, Sciences Faculty, University of Porto, Rua do Campo Alegre, 4169-007 Porto, Portugal; E-Mail: vmvascon@fc.up.pt; 3 Recursos Mariños e Pesquerías, Facultade de Ciencias, Universidade da Coruña, Alejandro de la Sota, no.1 C.P:15008 A Coruña–España, Spain; E-Mail: nufero@gmail.com (N.F.); 4 Escola Superior de Tecnologia da Saúde do Porto, Instituto Politécnico do Porto, Rua Valente Perfeito 322, 4400-330 Vila Nova de Gaia, Portugal; E-Mail: mrm@estsp.ipp.pt (R.F.M.); 5 IBMC–Institute for Molecular and Cell Biology, University of Porto, R. Campo Alegre 823, 4150-180 Porto, Portugal

**Keywords:** brackishwater cyanobacteria, sea urchin Paracentrotus lividus embryo larval bioassay, brine shrimp Artemia salina lethality test, benthic habitats

## Abstract

Cyanobacteria are a diverse group of Gram-negative bacteria that produce an array of secondary compounds with selective bioactivity against vertebrates, invertebrates, plants, microalgae, fungi, bacteria, viruses and cell lines. The aim of this study was to assess the toxic effects of aqueous, methanolic and hexane crude extracts of benthic and picoplanktonic cyanobacteria isolated from estuarine environments, towards the nauplii of the brine shrimp *Artemia salina* and embryos of the sea urchin *Paracentrotus lividus*. The *A. salina* lethality test was used as a frontline screen and then complemented by the more specific sea urchin embryo-larval assay. Eighteen cyanobacterial isolates, belonging to the genera *Cyanobium*, *Leptolyngbya*, *Microcoleus*, *Phormidium*, *Nodularia*, *Nostoc* and *Synechocystis,* were tested. Aqueous extracts of cyanobacteria strains showed potent toxicity against *A. salina,* whereas in *P. lividus,* methanolic and aqueous extracts showed embryo toxicity*,* with clear effects on development during early stages. The results suggest that the brackishwater cyanobacteria are producers of bioactive compounds with toxicological effects that may interfere with the dynamics of invertebrate populations.

## 1. Introduction

Cyanobacteria are an ubiquitous and promising group of prokaryotes from which novel bioactive compounds can be isolated [1]. Cyanobacteria are known for producing a broad array of secondary metabolites, including toxins. Chemically, they can be lipopeptides, amino acids, fatty acids, macrolides and amides with ecological, biological and pharmacological activity [2]. Studies concerning the bioactivity of cyanobacteria have been mainly directed to planktonic forms from marine and freshwater habitats, whilst estuarine originated cyanobacteria are mostly unexplored [1,2].

For cyanotoxin detection and biological activity screening in cyanobacteria, several bioassays have been used. The brine shrimp *Artemia salina* lethality test is usually used to study the effects of cyanobacteria in coastal environments such as estuarine, marine and hipersaline ecosystems. This bioassay provides a viable tool since *A. salina* is a representative species of the zooplankton community. Moreover, the brine shrimp bioassay is an inexpensive test and without ethical constraints [2–4].

The *A. salina* bioassay developed by Vanhaecke *et al.* [5–7] is a useful tool for preliminary biological and pharmacological activity analysis. *A. salina* is an organism occurring in brackish and marine waters, adaptable to large ranges of salinity (5 to 250 g L^−1^) and temperature (6 to 35 °C) [8]. Moreover, this organism is vital to the pelagic ecology of a coastal ecosystem (estuaries, bays, harbors and other nearshore environments). Although it is still considered the basic screening test for cyanobacteria from coastal environments, other sensitive and more specific screening bioassays have been applied, specifically the ones using embryos of invertebrates, viruses and cell lines [8,9]. For studies involving embryogenesis, one of the most described assays uses echinoids, namely sea urchins like *Paracentrotus lividus* species. This is a common species in European coastal habitats as well as being a vital organism in the benthic ecology of these systems. Furthermore, the sea urchin is an euryhaline organism like the brine shrimp *A. salina*. Sea urchins are commonly chosen as model organisms since they are known to be very common seashore organisms constituting up to 90% of the benthic biomass. Furthermore, compared with other employed invertebrates such as arthropods and mollusks, they occupy a key phylogenetic position (deuterostomes). Besides, sea urchins produce a great amount of eggs, feasible to be fertilized in sea water and to develop optically clear embryos among others characteristics, making these organisms good test models. Moreover, the early stages of these organisms are more sensitive to toxicants than the adults and microscopical measures are easy to perform [6,7,10]. All these noteworthy characteristics of these two model organisms enable their use in an estuarine study.

Although the information about cyanobacterial bioactive compounds has been increasing over the last decades, less information of estuarine as well as benthic species is available. The aim of this study was to evaluate the toxic effects of organic and aqueous crude extracts of benthic cyanobacteria isolated from estuaries of North and Centre of Portugal towards the nauplii of the brine shrimp *A. salina* and embryos of the sea urchin *P. lividus*.

## 2. Results and Discussion

The sampling resulted in the isolation of 18 cyanobacteria isolates from seven different genera, *Cyanobium*, *Leptolyngbya*, *Microcoleus*, *Phormidium*, *Nodularia*, *Nostoc* and *Synechocystis* of Douro, Minho and Vouga estuaries (Table 1). The isolates were collected from benthic and picoplanktonic habitats with salinity ranging from 5–35 g L^−1^.

The putative bioactivity of these cyanobacteria was evaluated against *A. salina* and the sea urchin *P. lividus*. In the *A. salina* lethality bioassay, the aqueous crude extracts of strains LEGE 07075, LEGE 07076, LEGE 06070 and LEGE 07074 were the most toxic. The LC_50_ values obtained for LEGE 07075, LEGE 07076 and LEGE 07074 were 8.95, 12.10 and 13.78 mg mL^−1^, respectively. The other extracts had LC_50_ values above 15 mg mL^−1^ (Table 2). The results suggest that the aqueous crude extracts are more toxic than the organic ones (hexane and methanolic). Indeed, none of the hexane extracts induced mortality above 7%, which was also observed for some methanolic extracts (data not shown). Even still, one of 18 methanolic extracts, LEGE 06071, induced acute effects on the *A. salina* nauplii with a LC_50_ of 17.81 mg mL^−1^ (95% confidence interval of 11.63 to 27.30 mg mL^−1^). It was also observed that the LC_50_ values calculated were more prominent at 48 h, which confirms the sensitivity of nauplii observed in previous works [3,8], suggesting that toxicity is much higher after 48 h exposure than after 24 h. Moreover, previous work showed that the crude extracts of marine *Synechocystis* strains reduced the survival of *A. salina* nauplii similarly to the results obtained here [11]. The most toxic extracts in this work were those of the filamentous forms *Leptolyngbya* and *Microcoleus*. These results may suggest that those genera are candidates for the production of a broad range of bioactive compounds in estuarine environments. A wide array of cyanobacterial bioactive metabolites is produced by large nonribosomal peptide enzymes, specifically synthetases and polyketide synthetases. From previous studies, it is known that the nonribosomal peptide enzyme genes are more abundant among the filamentous and heterocystous cyanobacteria [12,13].

**Figure 1:**
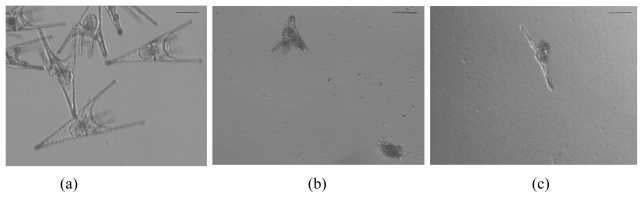
Effects of estuarine cyanobacterial extracts on embryonic development of sea urchin *P. lividus* after 48 h of exposure (a) Normal pluteus larva from the control treatment. (b) Abnormal four arms larva (with equal sized arms and shorter larval length). (c) Abnormal larva with only one-arm developed after exposure to cyanobacterial extracts. The bar represents 100 μm.

In the *P. lividus* bioassay, teratogenic effects at the pluteus larva stage and development inhibition effects at early stages, were observed. When the fertilized sea urchin eggs were exposed to aqueous and 70% methanolic crude cyanobacterial extracts, morphological abnormalities were observed: specifically, delayed or arrested development in the different early embryonic stages, loss of cell aggregation, larval malformations (four arms not formed or short length) and decreased the larval growth (Figure 1**)**.

The results of the *P. lividus* embryo-larval toxicity bioassay after 48 h of incubation with cyanobacterial extracts revealed that in the control trial 96.7 ± 7.2% of the *P. lividus* fertilized eggs developed to normal pluteus larva with an average length of 471.46 ± 6.20 μm. Increasing the concentration of the extract from 1.56 to 25.0 mg mL^−1^ did not produce lethal effects for either extract, although we observed developmental arrest at early stages at 1.56 mg mL^−1^ up to the concentration of 25.0 mg mL^−1^ (Table 3). At the highest concentration of the aqueous extracts (25.0 mg mL^−1^), embryos reached the morula, blastula or gastrula stages. Nevertheless, the LEGE 06069, LEGE 06077 and LEGE 07091 extracts led to slower embryo-larval development with the morula the only stage observed for the series of concentrations. The same observations were made with the 70% methanolic extracts LEGE 06070 and 07074. For the 70% methanolic extracts, at the highest concentration, 72% induced delayed embryo development to the morula stage in contrast to only 33% of the aqueous extracts. These results may indicate that 70% methanolic extracts can induce more toxic effects than the aqueous ones.

**Figure 2:**
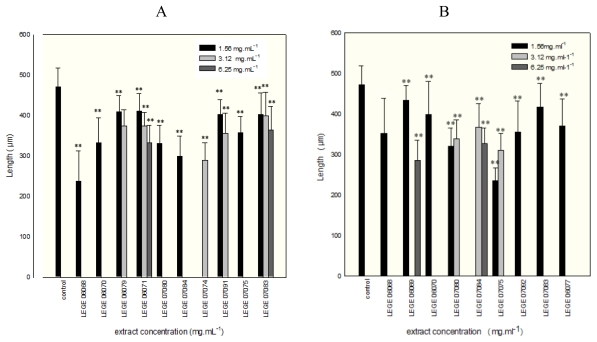
Mean values of length of normal *P. lividus* larvae (±SE) incubated on aqueous cyanobacterial extracts (A) and methanolic extracts (B), and on sea water as control. Asterisks indicate significant differences (** P ≤ 0.05) between extract and control.

Furthermore, an extract concentration dependent increase of toxic effects in *P. lividus* pluteus larva could be observed. Exceptions were LEGE 06069 and LEGE 07091 aqueous and LEGE 06079 methanolic extracts, which only led to the development of the morula stage independently of the extract concentration. Above 6.25 mg mL^−1^, for both aqueous and methanolic extracts, few sea urchin embryos could develop to pluteus stage. The embryos obtained from zygotes exposed to concentrations below 6.25 mg mL^−1^ of the aqueous extracts mainly developed 4four-arm pluteus larva with significant differences in larval length compared with the control (p < 0.05). For the LEGE 06079, LEGE 06071, LEGE 07080, LEGE 07084, LEGE 07074 and LEGE 07075 aqueous extracts, despite the larvae having normal development with four-arms, in all cases ANOVA showed that the larval lengths were significantly shorter (p < 0.05) than those of the control trial (Figure 2A and B).

For all the extracts and concentrations, significant differences were found (p < 0.05) from the control, excluding the pairs with concentrations of 1.6–3.2 mg mL^−1^ and 3.2–6.3 mg mL^−1^ of LEGE 07083 aqueous extract (p = 0.354 and 0.787, respectively). The results are in agreement with the results of Martins *et al.* [11] that also observed anomalies at the larval stage and inhibition of the embryogenesis exposed to marine cyanobacterial extracts, although the isolates now studied are from estuarine habitats. Our data demonstrate the presence of compounds in the extracts that can interfere with growth factors, as observed in previous studies [15]. Furthermore, we can also infer that estuarine cyanobacterial extracts have other compounds that probably inhibit the DNA synthesis and effect skeleton formation, since loss of cell aggregation was observed at morula stage as well as the inhibition of larval morphogenesis.

As already demonstrated for freshwater and marine cyanobacteria [16,17], the results obtained highlight the notion that cyanobacteria from estuarine habitats, specifically benthic forms, can be a prolific source for new bioactive compounds.

## 3. Experimental Section

### 3.1. Cyanobacteria sampling

**Figure 4:**
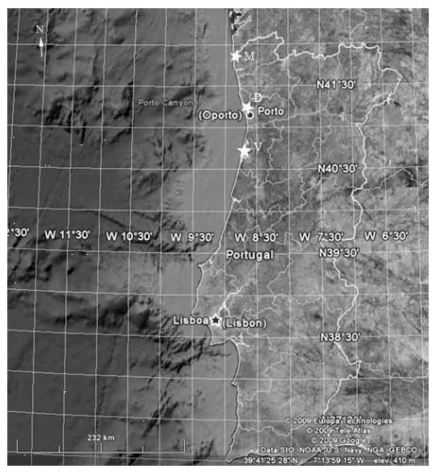
Geographic position of the sampling sites–Star symbols point out the sites: M-Minho estuary; D-Douro estuary and V-Vouga estuary.

Eighteen cyanobacterial strains were isolated from Portuguese Atlantic estuaries, specifically, Minho, Douro and Vouga, located for 42° 15′ to 40° 15′ north latitude and 8° 54′ to 8° 38′ west longitude (Figure 4). The samples were collected during the low tidal from benthos environments and water samples. Isolation and culture were performed in Z8 liquid medium [18] supplemented with NaCl (10 to 35 mg mL^−1^). Isolation procedure was done with use of the micromanipulation technique of single cells using both liquid and solid medium [19]. Cultures were grown under laboratory conditions at 25 °C, light intensity of 20.8–27.4 × 10^−6^ E m^−2^ s^−1^ and a light/dark cycle of 14/10 h. Cultures examined were unicyanobacterial and non-axenic.

### 3.2. Taxonomic characterization

Cyanobacterial strains were identified based on a morphological and molecular approach at the genus level. In regard to morphology, cells were analyzed by light microscopy and characterized according to Komárek and Anagnostidis [20,21] and Boone and Castenholz [22]. The molecular identification was based on 16S rRNA gene sequencing using primers and protocol previously described [23,24].

### 3.3. Preparation of extracts

For the preparation of the extracts, 6 to 8-week-old cultures were harvested by centrifugation, frozen, freeze-dried and stored at −20 °C. Freeze-dried cyanobacterial material was extracted three times for 20 seconds in ice with hexane (P.A. Sigma, USA), methanol (P.A. Sigma, USA), and distilled water for the *A. salina* bioassay, and with methanol 70% (v/v) and distilled water for sea urchin embryo-larval bioassay maintaining 1 hour in ice between each cycle of extraction. The supernatants collected by centrifugation (9300 *g* for 10 minutes) were evaporated and dissolved in artificial sea water (pH 8.0 ± 0.01). A 2-fold series of dilutions of each extract were made from an initial concentration of 100 and 25 mg dry weight. mL^−1^ to test with *A. salina* and *P. lividus*, respectively.

### 3.4. Preparation of the bioassays

The *A. salina* nauplii and the *P. lividus* embryo-larval bioassays were conducted in 96-well and 24-well microplates, respectively. Three replicates were performed for each treatment and control. Two negative controls were used, one only with artificial sea water and the other with the solvents used in the extractions. Solvents were evaporated and the residue dissolved in artificial seawater (ASW).

### 3.5. *A. salina* lethality assay

Dried cysts (JBL Novotemia, Germany) of *A. salina* were hatched in artificial sea water (1 g cyst L^−1^) at 25 °C under continuous illumination and aeration. After 24 h of incubation, the *A. salina* nauplii were collected and 10–20 individuals were transferred to each well. The assay consisted of the exposure of *A. salina* nauplii to a 2-fold series of dilutions of each extract. The toxicity was determined after 24 and 48 h of exposure at 25 °C in darkness. The number of dead larvae in each well was counted, as well as the total number of brine shrimps after Lugol’s fixation. Larvae were considered dead if no internal or external movement was observed during 30 seconds. Results are presented as percentage of mortality and LC_50_ values.

### 3.6. *P. lividus* embryo-larval toxicity assay

The embryo-larval bioassay was assessed by using embryos of the Mediterranean sea urchin *P. lividus.* Sea urchins were collected from wild populations on rocky shores at Valadares beach (41°05′ 30.37″N; 8°39′ 28.40″W). Adult *P. lividus* specimens were dissected and the gametes collected directly from the gonads [19]. *In vitro* fertilization was induced in ASW. Successful fertilization rate was determined by counting the fertilized eggs in 10 μL aliquots. This assay consisted of exposing *P. lividus* fertilized eggs to 3 mL of a series of dilutions of aqueous and 70% methanolic crude filtered cyanobacterial extracts (0.2 μm pore filter) and to controls (ASW) during 48 h at 20 °C. The maximum concentration used of each extract was 25 mg dry weight mL^−1^. In each well, 20 fertilized eggs per mL of solution were added. After 40% formalin fixation, the embryogenesis success was evaluated by measuring the pluteus larval percentage and length.

### 3.7. Statistical analyses

The data obtained from the *A. salina* bioassay were analyzed with the Probit analysis or (Trim) Spearman-Kaber depending on the number of partial mortalities. The endpoint determined was the LC_50_ values with 95% confidence intervals at 24 and 48 h. The results of sea urchin embryo-larval toxicity test are reported as the mean larval length ± standard deviation (SD). The goodness of fit of the data to normal distribution was test applying the Kolmogorov-Smirnov statistic. The statistical significance of the different means observed in the control and those in the extracts were determined by one-way analysis of variance (ANOVA). The statistical analysis was performed with SPSS software (version 17.0, USA) after the underlying assumptions of normality and homoscedasticity were checked. When those assumptions were not met, a non-parametric Kolmogorov-Smirnov test for two-independent samples was applied. A significant level of *p* < 0.05 was accepted.

## 4. Conclusions

In this study, 18 crude extracts of estuarine cyanobacteria isolates were assessed for toxic effects against the *A. salina* nauplii and the *P. lividus* sea urchin embryos. Among the cyanobacterial isolates, 78% are filamentous forms and the other 20% coccoid, with *Leptolyngbya* being the most dominant genus. Furthermore, the strains belonging to the genus *Leptolyngbya* were collected only from benthic environments, which underline that the benthic forms can be a good source of novel compounds, and also a putative risk to the benthic community.

The results show that aqueous crude extracts can induce more ecotoxicological effects on *A. salina* nauplii than the methanolic and lesser than hexane extracts. Regarding the sea urchin bioassay, the data obtained indicate teratogenic effects at the pluteus larva stage and toxic effects during early embryogenesis of *P. lividus* induced by the methanolic and aqueous crude extracts.

The data obtained may also suggest that estuarine cyanobacteria are producers of bioactive compounds of hydrophilic and hydrophobic nature. The presence of hydrophilic compounds in the extracts can pose a putative ecological threat to these environments, since these compounds become more available after cell lysis or when released to the environment. Still, the putative different nature of the compounds can be seen also as potential source of new pharmacological compounds.

The outcome of this work allowed us to reinforce the notion that is important to increase the research on estuarine cyanobacteria, with further characterization of their bioactive compounds.

## Figures and Tables

**Figure 3:**
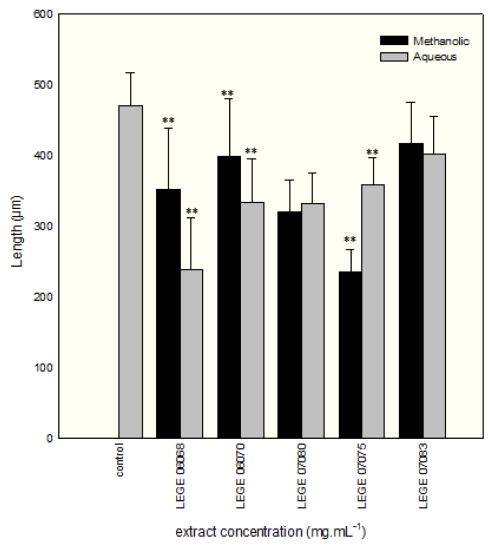
Comparison of the mean values of length of normally developed *P. lividus* larvae (±SE) incubated on cyanobacterial methanolic extracts and on sea water control to a concentration of 1.6 mg mL^−1^. Asterisks indicate significant differences (** P ≤ 0.05) between extract and control.

**Table 1:** Cyanobacterial isolates included in the study and their geographic position.

	Identification	Coordinates	

Lab code	Taxon	Latitude	Longitude
**LEGE 06068**	*Cyanobium* sp.	41° 8′50.77″N	8°39′12.89″W
**LEGE 06069**	*Leptolyngbya* sp.	41° 8′50.45″N	8°38′2.13″W
**LEGE 06070**	*Leptolyngbya* sp.	41° 8′50.45″N	8°38′2.13″W
**LEGE 06071**	*Nodularia* sp.	40°38′32.87″N	8°39′47.85″W
**LEGE 06072**	*Phormidium* sp.	40°40′16.42″N	8°43′24.36″W
**LEGE 06078**	*Phormidium* sp.	41° 8′12.20″N	8°39′54.65″W
**LEGE 06079**	*Synechocystis* sp.	41° 8′12.20″N	8°39′54.65″W
**LEGE 07073**	*Synechocystis* sp.	40°40′16.42″N	8°43′24.36″W
**LEGE 07074**	*Leptolyngbya* sp.	41° 8′48.17″N	8°39′38.79″W
**LEGE 07075**	*Leptolyngbya* sp.	41° 8′50.45″N	8°38′2.13″W
**LEGE 07076**	*Microcoleus* sp.	41°54′5.00″N	8°48′51.88″W
**LEGE 07077**	*Nostoc* sp.	41°52′40.13″N	8°50′6.33″W
**LEGE 07080**	*Leptolyngbya* sp.	41°52′2.50″N	8°51′35.90″W
**LEGE 07083**	*Synechocystis* sp.	41° 8′48.17″N	8°39′38.79″W
**LEGE 07084**	*Leptolyngbya* sp.	41°52′16.76″N	8°50′39.66″W
**LEGE 07085**	*Leptolyngbya* sp.	41° 8′50.77″N	8°39′12.89″W
**LEGE 07091**	*Leptolyngbya* sp.	40°40′16.42″N	8°43′24.36″W
**LEGE 07092**	*Microcoleus* sp.	40°40′16.42″N	8°43′24.36″W

**Table 2:** 24-h and 48-h LC_50_ values (with 95% confidence limits) for *A. salina* exposed to 18 estuarine cyanobacterial methanolic and aqueous extracts.

	LC_50_ (95% confidence limits ) mg mL^−1^							

	Methanolic extracts				Aqueous extracts			
Code	24 h		48 h		24 h		48 h	
**LEGE 06068**	-	-	-	-	-	-	79.92	(36.28–653.08)
**LEGE 06069**	-	-	-	-	-	-	32.62	(27.22–39.10)
**LEGE 06070**	-	-	-	-	-	-	15.42	(5.00–27.56)
**LEGE 06071**	-	-	17.81	(11.63–27.30)	-	-	53.20	(37.97–65.94)
**LEGE 06072**	-	-	-	-	-	-	20.13	(12.18–30.36)
**LEGE 06077**	-	-	-	-	72.11	(54.96–94.60)	72.01	(57.88–86.61)
**LEGE 06078**	-	-	-	-	-	-	28.60	(8.93–40.58)
**LEGE 06079**	-	-	-	-	22.63	NR	-	-
**LEGE 07073**	-	-	-	-	-	-	-	-
**LEGE 07074**	-	-	-	-	-	-	13.78	(11.46–16.55)
**LEGE 07075**	-	-	-	-	-	-	8.95	(4.98–15.00)
**LEGE 07076**	-	-	-	-	-	-	12.10	(9.95–14.64)
**LEGE 07080**	-	-	-	-	-	-	-	-
**LEGE 07083**	-	-	-	-	-	-	-	-
**LEGE 07084**	-	-	-	-	32.62	(28.00–38.00)	32.62	(28.00–38.00)
**LEGE 07085**	-	-	-	-	94.9	(73.14–133.15)	64.61	(52.23–79.92)
**LEGE 07091**	-	-	-	-	26.15	(19.85–34.4)	26.15	(19.85–34.4)
**LEGE 07092**	-	-	-	-	20.68	(15.17–28.18)	18.08	(14.15–23.10)

“-“: values not determined since the mortality (data not shown) was less than 50%; NR: (not reliable).

**Table 3:** Embryonic development of *P. lividus* after 48 h exposure to different concentrations (25.0 to 1.56 mg mL^−1^) of aqueous and methanolic crude extracts of estuarine cyanobacterial isolates.

	Aqueous					Methanolic				

Code	25.00	12.50	6.25	3.12	1.56	25.00	12.50	6.25	3.12	1.56
**LEGE 06068**	G	G	G	P	P	M	M	M/G	M/P	G/P
**LEGE 06069**	M	M	M	M	M	M	M	P/L	G	M/P/L
**LEGE 06070**	B	B	B	G/P	G/P/L	M	M	M	M/G	M/G/L
**LEGE 06078**	B	B	B	B	G/P	G	G/P	G/P	G/P	G/P
**LEGE 06079**	M	G	P	L	L	M	M	M	M	M/L
**LEGE 06071**	G	P	L	L	L	B	B	B	B	B
**LEGE 06072**	G	G	G/P	G/P	P	M	M/B	G	G	P
**LEGE 07080**	M	M	M	M/G/L	M/G/L	B	G	G/P	L	L
**LEGE 07084**	B	B	B	B	G/P/L	G	G	P/L	L	L
**LEGE 07074**	B	B	B	G	G/P/L	M	M	M	P/L	M/G/L
**LEGE 07085**	B	B	B	B	G	B	B	G	P	P
**LEGE 07075**	G	G	G	P	L	M	B	P	G	P/L
**LEGE 07091**	M	M	M	M	M	M	M	G	P/L	L
**LEGE 07076**	B	B	B/G	B/G	G	M	M	M	G	M/G/L
**LEGE 07092**	G	G	G	G	G	M	M	M/G	G/P	G/L
**LEGE 06077**	M	M	M	M	G/P	M	M	M/P	P	P/L
**LEGE 07073**	B	B	B	G	G	M	M	M	P	M/P/L
**LEGE 07083**	M	G/P	P/L	L	L	M	B	B	B/G/P	L

M-morula, B-blastula, G-gastrula, P-prism and L-pluteus larva.

## References

[1] Singh S Kate BN Banerjee UC Bioactive compounds from cyanobacteria and microalgae: an overview Crit Rev Biotechnol 2005 25 73 95 16294828 10.1080/07388550500248498

[2] Jaiswal P Singh PK Prasanna R Cyanobacterial bioactive molecules--an overview of their toxic properties Can J Microbiol 2008 54 701 717 18772933 10.1139/w08-034

[3] Sánches-Fortún S Sanz S Barahona MV Acute toxicity of several organophosphorous insecticides and protection by cholinergic antagonist and 2-PAM on *Artemia salin*a larvae Arch Environ Contam Toxicol 1996 31 391 398 8854833 10.1007/BF00212678

[4] Caldwell GS Bentley MG Olive PJW The use of a brine shrimp (*Artemia salina*) bioassay to assess the toxicity of diatom extracts and short chain aldehydes Toxicon 2003 42 301 306 14559082 10.1016/s0041-0101(03)00147-8

[5] Vanhaecke P Persoone G Claus C Sorgeloos P Proposal for a short-term toxicity test with Artemia nauplii Ecotoxicol Environ Saf 1981 5 382 387 7297475 10.1016/0147-6513(81)90012-9

[6] Sleet RB Brendel K Brine shrimp, *Artemia salina*: a potential screening organism for initial teratology screening Proc West Pharmacol Soc 1983 26 169 170 6889334

[7] Carballo JL Hernandez-Inda ZL Perez P Garcia-Gravalos MD A comparison between two brine shrimp assays to detect *in vitro* cytotoxicity in marine natural products BMC Biotechnol 2002 2 17 12270067 10.1186/1472-6750-2-17 PMC130034

[8] Nunes BS Carvalho FD Guilhermino LM Van Stappen G Use of the genus Artemia in ecotoxicity testing Environ Pollut 2006 14 453 462 16677747 10.1016/j.envpol.2005.12.037

[9] Krueger RJ Book Review of Bioactive Natural Products. Detection, Isolation, and Structural Determination. Second Edition J Med Chem 2008 51 3659 3659

[10] Boudouresque CF Verlaque M Ecology of *Paracentrotus lividus* Edible Sea Urchins: Biology and Ecology Lawrence JM Elsevier publ. Amsterdam, The Netherlands 2007 243 285

[11] Martins R Fernandez N Beiras R Vasconcelos V Toxicity assessment of crude and partially purified extracts of marine *Synechocystis* and *Synechococcus* cyanobacterial strains in marine invertebrates Toxicon 2007 50 791 799 17686503 10.1016/j.toxicon.2007.06.020

[12] Ehrenreich IM Waterbury JB Webb EA Distribution and diversity of natural product genes in marine and freshwater cyanobacterial cultures and genomes Appl Environ Microbiol 2005 71 7401 7413 16269782 10.1128/AEM.71.11.7401-7413.2005 PMC1287672

[13] Alexandra AR Janine NC Mohamed AM Brett AN The *Synechocystis* sp. PCC6803 Sfp-Type Phosphopantetheinyl Transferase Does Not Possess Characteristic Broad-Range Activity ChemBioChem 2009 10 1869 1877 19562788 10.1002/cbic.200900095

[14] Sugni M Mozzi D Barbaglio A Bonasoro F Candia Carnevali MD Endocrine disrupting compounds and echinoderms: New ecotoxicological sentinels for the marine ecosystem Ecotoxicology 2007 16 95 108 17253161 10.1007/s10646-006-0119-8

[15] Da Rocha AB Lopes R Schwartsmann G Natural products in anticancer therapy Curr Opin Pharmacol 2001 364 369 11710734 10.1016/s1471-4892(01)00063-7

[16] Wiegand C Pflugmacher S Ecotoxicological effects of selected cyanobacterial secondary metabolites: A short review Toxicol Appl Pharmacol 2005 203 201 218 15737675 10.1016/j.taap.2004.11.002

[17] Mundt S Kreitlow S Nowotny A Effmert U Biochemical and pharmacological investigations of selected cyanobacteria Int J Hyg Environ Health 2001 203 327 334 11434213 10.1078/1438-4639-00045

[18] Kotai J Instructions for Preparation of Modified Nutrient Solution Z8 for Algae Norwegian Institute for Water Research Blindern, Oslo, Norway 1972 5

[19] Ripka R Isolation and purification of cyanobacteria Methods Enzymol 1988 167 3 28 3148836 10.1016/0076-6879(88)67004-2

[20] Komárek J Anagnostidis K Cyanoprokaryota, Part 1: Chroococcales, Süsswasserflora von Mitteleuropa, Bd 19/1 Gustav Fischer Verlag Stuttgart, Germany 1998 548

[21] Komárek J Anagnostidis K Cyanoprokaryota, Part 2: Oscillatoriales, Süsswasserflora von Mitteleuropa, Bd19/2 Elsevier/Spektrum Akademischer Verlag Heidelberg, Germany 2005 759

[22] Boone DR Castenholz RW Bergey’s Manual of Systematic Bacteriology Garrity GM Springer-Verlag New York, NY, USA 2001 1 600

[23] Jungblut A.-D Neilan BA Molecular identification and evolution of the cyclic peptide hepatotoxins, microcystin and nodularin, synthetase genes in three orders of cyanobacteria Arch Microbiol 2006 185 107 114 16402223 10.1007/s00203-005-0073-5

[24] Neilan BA Jacobs D Del Dot T Blackall LL Hawkins PR Cox PT Goodman AE rRNA sequences and evolutionary relationships among toxic and nontoxic cyanobacteria of the genus Microcystis Int J Syst Bacteriol 1997 47 693 697 10.1099/00207713-47-3-693 9226902

[25] Fernández N Beiras R Combined toxicity of dissolved mercury with copper, lead and cadmium on embryogenesis and early larval growth of the *Paracentrotus lividus sea*-urchin Ecotoxicology 2001 10 263 271 11556113 10.1023/a:1016703116830

